# Positive effects of parent–child group emotional regulation and resilience training on nonsuicidal self-injury behavior in adolescents: a quasi-experimental study

**DOI:** 10.3389/fpsyt.2024.1343792

**Published:** 2024-03-20

**Authors:** Junxiang Cheng, Juan Zhao, Baoli Song, Hong Han, Na Liu, Yangjie Chen, Xiaomei Liu, Yue Dong, Weina Bian, Zhifen Liu, Shifan Han

**Affiliations:** ^1^ Department of Psychiatry, the First Hospital of Shanxi Medical University, Taiyuan, China; ^2^ School of Nursing, Shanxi Medical University, Taiyuan, China; ^3^ Department of Magnetic Resonance Imaging, Shanxi Bethune Hospital, Taiyuan, China; ^4^ Department of Orthopedics, The First Hospital of Shanxi Medical University, Taiyuan, China; ^5^ Department of Intensive Care Unit (ICU), the Affiliated Lianyungang Hospital of Xuzhou Medical University, Xuzhou, China; ^6^ Department of Nursing, Hanzhong Central Hospital, Hanzhong, China

**Keywords:** adolescent, emotional regulation, nonsuicidal self-injury, parent-child relation, resilience

## Abstract

**Background:**

Nonsuicidal self-injury (NSSI) among adolescents is a growing global concern. However, effective interventions for treating NSSI are limited.

**Method:**

A 36-week quasi-experimental study design of parent–child group resilience training (intervention group) for adolescents aged 12–17 years was used and compared with treatment-as-usual (control group). The primary endpoint was the frequency of NSSI assessed with the Ottawa Self-Injury Inventory (OSI), and the secondary endpoints were the levels of depression, hope, resilience, and family adaptability and cohesion as assessed by the 24-item Hamilton depression rating scale (HAMD-24), Herth Hope Scale (HHS), Connor-Davidson Resilience Scale (CD-RISC), and Family Adaptability and Cohesion Evaluation Scale, second edition (FACES-II-CV), respectively.

**Result:**

A total of 118 participants completed the trial. Both groups showed a significant reduction in NSSI frequency after 12, 24, and 36 weeks of intervention (*p*< 0.05), although the intervention group did not differ significantly from the control group. After 12, 24, and 36 weeks of intervention, the CD-RISC, HHS, HAMD-24, and FACES-II-CV scores in the intervention and control groups improved over baseline (*p*< 0.05). Furthermore, the intervention group had higher scores on the CD-RISC, HHS, and FACES-II-CV and lower scores on the HAMD-24 than the control group after 12, 24, and 36 weeks of intervention (*p  <* 0.05).

**Conclusion:**

Parent–child group emotional regulation and resilience training showed promise as treatment options for NSSI among adolescents, leading to increased hope, resilience, and improved family dynamics among NSSI teens. Moreover, NSSI frequency significantly decreased in the intervention group compared to baseline.

## 1 Introduction

Nonsuicidal Self-injury (NSSI) refers to the act of intentionally injuring one’s own body through scratching, cutting, biting, or scalding without suicidal intention (1). A study conducted by the Centers for Disease Control (CDC) in the USA revealed that the prevalence of NSSI typically peaks during adolescence, around the ages of 15–16, with a rate of 15.3% (2). Zhang et al. reported that at least one instance of NSSI occurred in 21.7% of individuals and that its onset occurred earlier in females compared to in males (3). Although NSSI and suicidal behavior are different diagnoses in the Diagnostic and Statistical Manual of Mental Disorders Fifth edition (DSM-V), studies have found that non-suicidal self-injury is often a precursor to suicide and is considered a significant risk factor for suicide (4–6). The high prevalence of NSSI and its association with attempted and completed suicides impose a substantial financial burden on families and societies, making it a pressing public health concern (7).

The onset of NSSI in adolescents is a complex phenomenon influenced by multiple factors, including psychological factors, problematic parental relationships, adverse childhood experiences, rumination, and sleep disorders (8–11). Parent–child relationships play a crucial role in children’s development at all stages. The biosocial model posits that early vulnerability and family environment risk factors may contribute to more severe emotional and behavioral dysregulation, such as NSSI (12, 13). A longitudinal cohort study showed that poor-quality attachment to parents could predict the frequency of NSSI (14). Findings from China also demonstrated that poor parent–child relationships are a high-risk factor for adolescent NSSI (11, 15). NSSI not only affects adolescents themselves, but it also has a substantial impact on the entire family. Parents constantly navigate a delicate balance and are unaware of words or events that might trigger their children’s self-harming behavior. This constant state of tension can lead to “empathetic burnout,” where parents struggle to respond with compassion. Moreover, the negative emotions experienced by adolescents in response to their parents’ reactions can further exacerbate their self-injurious behavior (16). The human birth theory suggests that defects in the mother-newborn relationship from birth to one year of age may be associated with the development of mental illness later in adolescence. If the needs of the newborn’s development and the relationship with caregivers are not met, this hidden deficiency may lead to mental disordor in the child during the puberty (17). Therefore, improving the quality of parent–child relationships is an essential factor in preventing self-injurious behavior in adolescents.

A study found a negative association between adolescent resilience and NSSI (18). Resilience is a multidimensional and dynamic process that encompasses the development of a sense of purpose or meaning in life, positive emotions, self-esteem, positive coping strategies, optimism, social support, and cognitive flexibility (19, 20). It is a positive personality trait that enables individuals to effectively deal with adverse and stressful situations and can be enhanced through training (21). A high level of resilience indicates strong coping abilities and adaptability, enabling individuals to maintain a positive perspective and effectively navigate future adversities (11, 18). Therefore, resilience training is a viable and potentially valuable intervention for promoting patient well-being.

Stress management and resilience training (SMART) was set up by health managers Werneburg et al. at the Mayo Clinic in Rochester, USA (22). Previous studies have shown that SMART training improves resilience among medical practitioners, breast cancer patients after surgery, and patients with major depressive disorder, reducing their anxiety, depression, and stress levels (22–24). However, to the best of our knowledge, no study has reported the effects of SMART on NSSI in adolescents. Herein, we hypothesized that parent–child emotional regulation and resilience group training conducted by professional psychotherapists based on SMART might reduce the frequency of adolescent NSSI behavior by improving the parent–child relationship and resilience.

## 2 Methods

### 2.1 Participants

Adolescent inpatients were recruited from the Mental Health Department of the First Hospital of Shanxi Medical University between April 2020 and September 2021. The inclusion criteria were as follows: (1) engaging in NSSI for at least five days during the previous six months and at least once during the past month, and (2) aged 12–17 years old. The exclusion criteria were as follows: (1) acute psychotic symptoms, acute intent to self-harm, or suicidal thoughts or behaviors; (2) inability of parents or patients to cooperate with 12 weeks of training therapy; (3) diagnosis of other somatic or psychotic diseases; (4) receiving non-convulsive electroconvulsive therapy (MECT) within the past two months; and (5) parents with a previous or present diagnosis of mental illness. The recommended patients and their families were informed about the treatment and signed informed consent forms if they agreed to participate. Both patients and their parents signed informed consent forms, and the study was reviewed and approved by the Ethics Committee of the First Hospital of Shanxi Medical University (ethics number: k-k159). A total of 132 adolescents were admitted and assigned numbers at the time of recruitment, with odd numbers assigned to the intervention group and even numbers assigned to the control group, maintaining a 1:1 allocation ratio. Two experienced clinical psychologists, who were blinded to the participant’s group allocation, conducted assessments and the Kappa value for the consistency test of scale assessment was 0.82 (p > 0.05).

### 2.2 Study design

The present study utilized a quasi-experimental design to compare the effects of parent–child group emotional regulation and resilience training with treatment as usual (TAU) on adolescent NSSI.

### 2.3 Procedure

**Figure 1:**
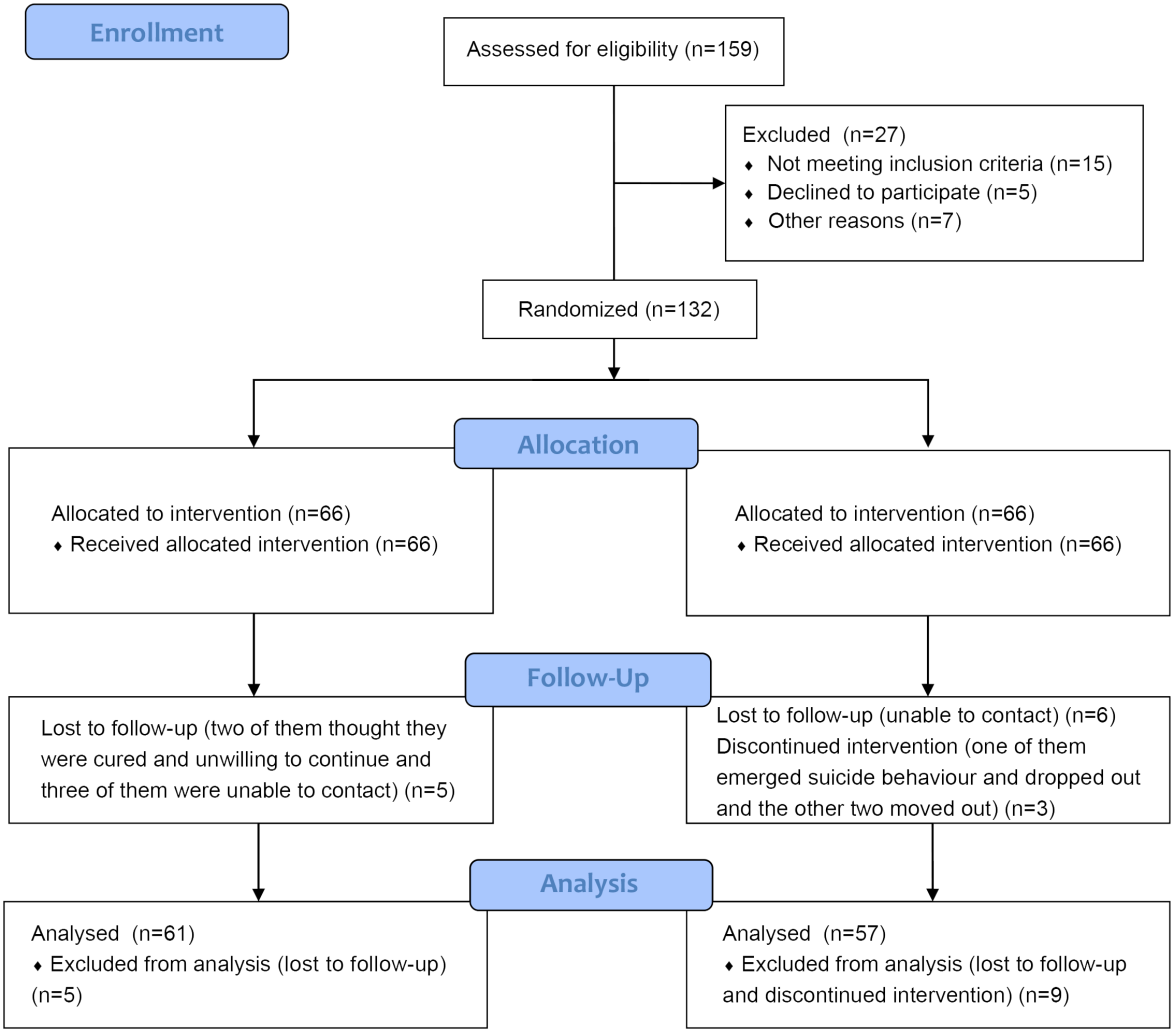
Enrollment flow diagram.

Participants were recruited based on recommendations from psychiatrists, residents, and nurses in the psychiatric ward (**Figure 1**). The investigators conducted baseline surveys and evaluations, and both groups completed a second assessment 12 weeks after enrollment; a third assessment was performed at 24 weeks, and a fourth assessment at 36 weeks. Psychotherapy was used in both groups of participants.

Brent and Kolko defined “psychotherapy as a modality of treatment in which the therapist and patient(s) work together to ameliorate psychopathologic conditions and functional impairment through focus on the therapeutic relationship; the patient’s attitudes, thoughts, affect, and behavior; and social context and development” (25). Although there are various clinical psychotherapy techniques, they are generally divided into individual therapy, group therapy, and family therapy according to the population. Polese et al. found that group therapy was effective in interventions for psychiatric disorders even in young patients suffering from psychosis who were resistant to pharmacotherapy (26).

### 2.4 Intervention group

Patients in the intervention group received parent–child group emotional regulation and resilience training conducted by the same psychotherapist and assistant in each session. The therapists in the intervention group were qualified psychotherapists with 10 years of experience, and the assistant was a graduate student in psychology. The intervention group was carried out in a group form and was divided into 11 closed groups, with each group consisting of 12 people, including six patients and their mothers or fathers. The training consisted of 12 weekly sessions, each lasting 80 minutes. The training schedule was agreed upon by the patients and their parents, and the training time per week was fixed for each group. The entire treatment, including the post-discharge phase, required discussions with parents and children to ensure adherence to the training schedule. Considering adolescents’ psychological characteristics, the training content integrated emotional regulation strategies and resilience training and focused on four areas: awareness of the propensity for nervous system stress, attention training, learning the five core principles of increased emotional resilience (gratitude, compassion, acceptance, meaning, and forgiveness), and relaxation training (**Table 1**).

**Table 1:** Parent-child group emotional regulation and resilience training content.

week	Theme	Skill training	Conduct contents
1	Analysis of stressors	Perceived personal sources of stress	1.get to know each other (Self-introduction of group members) 2.stress, stressors, how to perceive stress, stress coping discussion 3.assignment: daily stress log
2	Attention training	New detail discovering	1.practice feedback (group discussion) 2.take advantage of changes in the setting of the treatment room to allow participants to notice the differences (sharing and dicussion) 3.meditation practice 4.assignment: recording a positive discovery per day
3	Gratitude	Say thank you to the five most important people in your life	1.assignment feedback (group discussion) 2.listing five important people in life 3.saying “thank you” to them (on-site calls) 4.assignment: Daily gratitude journal
4	Compassion	Blessing expression	1.assignment feedback (group discussion) 2.blessings from strangers 3.blessings for others 4.assignment: Daily blessing journal
5	Acceptance	Relaxation technique	1.assignment feedback (group discussion) 2.safty island 3.abdominal breathing training 4.assignment: doing relaxation exercises every day before bed
6	Meaning	Meaning and purpose of life	1.practise feedback 2.exploring the purpose of "being alive" 3.group discussion “my own value” 4.assignment: doing relaxation exercises every day before bed
7	Forgiveness	Forgiving the others	1.practise feedback 2.an "unforgivable person" in my life - aware of the source of my anger 3.think about the problem from a different perspective meditation relaxation practice 4.assignment:5-10 minutes’Meditation relaxation practice every day before bed
8	Attachment relationships	Expectations and gains in interpersonal communication	1.practise feedback 2.discussion of positive and negative feelings in relationships 3.expectations for the identified problem 4.assignment:5-10 minutes’Meditation relaxation practice every day before bed
9	Mindfulness meditation	Mindfulness meditation skill	1.practise feedback 2.explore difficult mindfulness practices 3.assignment: practicing the mindfulness meditation every day before bed
10	Patience and anger	Don't try to change others	1.practise feedback 2. exceptions finding- your ideas test 3.event-emotion-thought pattern training 4.assignment: Daily Event-emotion-thought journal
11	Parent-child communication	How to express yourself	1.group discussion of daily journal 2.role reversal for communication 3.the group discussion of how they felt after the roles were reversed 4.assignment: dealing with a conflict through role reversal and recording the feeling
12	Summing-up	How to keep practicing	1.assignment feedback: group discussion 2.programme satisfaction survey 3.practice what you learned 4.thankful for yourself

During the sessions, participants were asked to practice in life scenarios and record the difficulties in daily practice for discussion. Each session included daily practice feedback on the previous and current training assignments. The day before each treatment, the assistant called the patients and parents to remind them of the upcoming training session to ensure their adherence.

### 2.5 Control group

The control group received routine treatment according to the psychiatrists’ treatment regimens, which commonly included weekly individual or group psychotherapy. To minimize the intervention bias in both groups, the control group required a minimum of 12 treatments. The psychotherapy content in the control group was not restricted and determined by the therapists. Psychotherapists in both groups received regular supervision. To minimize information bias among family members of patients in both groups, parents in both groups were required to attend the first session on “understanding NSSI.”

### 2.6 Research tools

Patients’ demographic data were gathered through a semi-structured interview developed for this study. Sociodemographic data included gender, age, educational level, and family structure. Clinically relevant variables included the Ottawa Self-Injury Inventory (OSI), 24-item Hamilton Depression Rating Scale (HAMD-24), Herth Hope Scale (HHS), Connor-Davidson Resilience Scale (CD-RISC), and Family Adaptability and Cohesion Evaluation Scale, second edition (FACES-II-CV).

The Ottawa Self-Injury Inventory (OSI) is a self-report questionnaire used to assess NSSI across multiple dimensions, including self-harm frequency in the past 1, 6, and 12 months, as well as its functions, alternative coping strategies, and potential addictive features. In this study, the item “How often in the past month have you injured yourself without the intention to kill yourself?” was used to investigate the frequency of self-injury. The Cronbach’s α coefficient of the Chinese version of this questionnaire was 0.952 (3).

The HAMD-24 (27) Chinese version is a commonly used clinical depression assessment scale that assesses the severity of depression. The Cronbach’s α coefficient of the HAMD-24 in the present study was > 0.8, while test-retest reliability was > 0.7, indicating good internal consistency and stability across different studies.

The Herth Hope Scale (HHS) is a tool for evaluating patients’ hope and consists of 12 questions scored on a 4-point Likert scale. The total score ranged from 12 to 48 points (28), with a higher score indicating a higher level of hope, which may predict better outcomes for patients with psychological health issues (29).

The Connor-Davidson Resilience Scale (CD-RISC) is a concise self-assessment tool with 25 questions; a higher score indicates a higher level of resilience. The Cronbach’s α coefficient for the Chinese version was 0.97 (30). The reliability and validity of this scale have been demonstrated in community samples, primary care outpatients, and psychiatric outpatients (31).

The Chinese version of the Family Adaptability and Cohesion Evaluation Scale, second edition (FACES-II-CV) was used to assess family function, and it has shown good validity and reliability in different studies (32, 33). Adaptability refers to a family’s ability to adjust to problems that arise at different stages of development, whereas cohesion refers to the emotional connection between family members; the higher the score, the better the family’s adaptability and cohesion.

### 2.7 Statistical analysis

SPSS 26.0 was used for data analysis. All analyses were full analysis sets (FAS). Based on Cohen’s guidelines, with an expected medium effect in the intervention group (f = 0.25), power (1-β) set at 0.8, and α = 0.05, a minimum of 64 participants were needed in each group. Quantitative data following a normal distribution were expressed as mean ± SD, and intergroup comparisons were conducted using t-tests. Quantitative data that did not conform to a normal distribution were expressed as median (P25, P75), and intergroup comparisons were conducted using the Mann-Whitney U test. Numerical data were expressed as rates, and the chi-square test or Fisher’s exact test was used for intergroup comparisons. Repeated measures analysis of variance (ANOVA) was used to test the effects of group (intervention vs. control) and time (baseline, 12 weeks, 24 weeks, and 36 weeks) on the CD-RISC, HHS, HADM-24, and FACES-II-CV scores. The main effects of group and time, as well as their interaction effect, were examined.

To account for the potential correlation among the self-injury outcomes from repeated measures at baseline and post-test, data were analyzed using generalized estimating equations. Generalized estimating equations (GEE) also estimated the working correlation parameters for the total sample and detected differences between the intervention and control groups. The GEE models incorporated baseline data and changes in outcomes over time across different groups. The significance level was set at *p*< 0.05.

## 3 Results

### 3.1 Participant characteristics

A total of 132 eligible participants were enrolled in the study and randomly allocated to the intervention (n = 66) or control (n = 66) group. The reasons for dropping out included being unable to contact (n = 9), not wanting to continue believing they were cured (n = 2), experiencing suicidal behavior and dropping out of the study (n = 1), and stopping intervention (n = 2). A total of 61 participants remained in the intervention group and 57 in the control group at the 36-week follow-up, with a follow-up completion rate of 89.4% (**Figure 1**).

**Table 2** presents the patients’ demographic and clinical characteristics. The mean age of the subjects was 15.00 ± 1.00 years. The intervention group included 21 men and 40 women, and the control group comprised 25 men and 32 women. No significant differences were found in terms of gender, age, education level, or family structure between the intervention and control groups at baseline (p > 0.05).

**Table 2:** intervention group and control group demographic characteristic.

Groups	Gender		Age	Educational level			Family structure	
	Male	Female		Primary school	junior high school	High school	Only child	Children with siblings
Control group	25	32	15(14,16)	3	36	18	40	17
Intervention group	21	40	15(14,15)	1	34	26	43	18
*χ^2^*/*z*	1.102		-1.036	2.303			0.001	
*P*	0.294		0.300	0.312			0.970	
Total	46	72	15.00±1.00	4	70	44	83	35

### 3.2 Clinical outcomes

**Figure 2:**
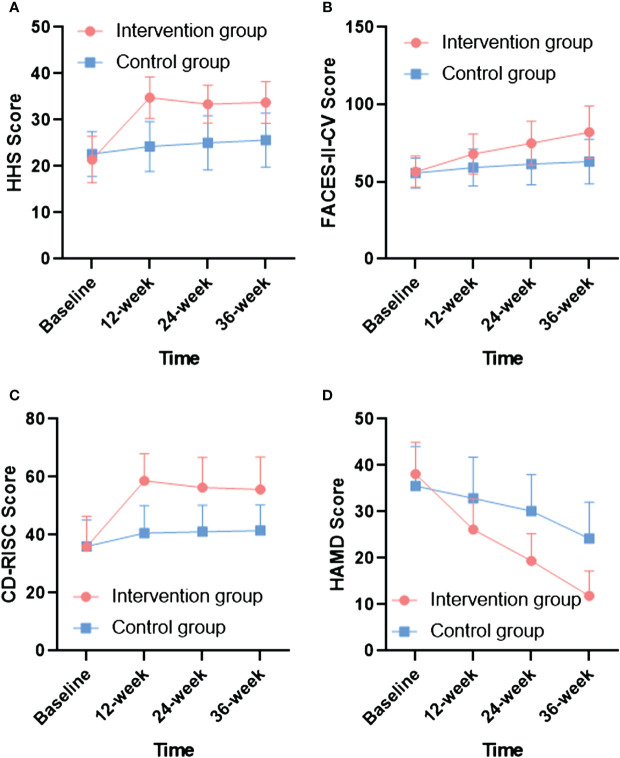
Comparison of HHS, FACES-II-CV, CD-RISC and HAMD-24 scores for intervention and control groups during different intervention times. **(A)** The changes of HHS scores in different intervention times; **(B)** The changes of FACES-II-CV scores in different intervention times; **(C)** The changes of CD-RISC scores in different intervention times; **(D)** The changes of HAMD-24 scores in different intervention times.

No significant difference was observed in each score between the two groups at baseline (p  > 0.05). After 12, 24, and 36 weeks of intervention, the CD-RISC, HHS, HAMD-24, and FACES-II-CV scores in the intervention and control groups improved over baseline. As shown in **Figure 2**, at 12 weeks of intervention, the CD-RISC and HHS scores of the intervention group reached their highest values, the HADM scores decreased with the increase in training time, and the FACES-II-CV scores improved with increasing training time. At the end of the intervention, the experimental group showed significant improvement compared to the control group, which suggests that the effects of the intervention were sustained during subsequent follow-up visits. The scores of the control group improved to some extent over time. Furthermore, the total CD-RISC, HHS, and FACES-II-CV scores were higher in the intervention group than in the control group after 12, 24, and 36 weeks of intervention (p  < 0 .05 for all categories). Similarly, the HAMD-24 scores in the intervention group were lower than those in the control group after 12, 24, and 36 weeks of intervention (p< 0.05, **Table 3**). Moreover, the main effect of group (intervention vs. control) on the CD-RISC, HHS, HADM-24, and FACES-II-CV scores was significant, as was the effect of time (baseline, 12, 24, and 36 weeks). The interaction effect of group and time was also significant, which suggests that the intervention had different effects on the outcomes over time (**Table 3**).

**Table 3:** Analysis results of repeated measurement (CD-RISC, HHS, HAMD, FACES-II-CV).

	Factors	sample	Time				*F*		
			Baseline	12W	24W	36W	Intervention	Time	Interaction
CD-RISC	Intervention Group	61	35.90±10.5	58.68±9.37^bc^	56.34±10.37^bcd^	55.72±11.21^bcd^	49.79^a^	283.94^a^	109.44^a^
	Control group	57	36.00±9.17	40.66±9.53^b^	41.08±9.12^b^	41.51±8.83^b^			
HHS	Intervention Group	61	21.42±4.99	34.80±4.48^bc^	33.38±4.05^bc^	33.78±4.50^bc^	58.64^a^	284.60^a^	139.07^a^
	Control group	57	22.65±4.86	24.28±5.40^b^	25.07±5.83^b^	25.64±5.82^bd^			
HAMD	Intervention Group	61	38.16±6.79	26.20±7.59^bc^	19.38±5.91^bcd^	11.84±5.40^bcde^	30.85^a^	627.19^a^	112.54^a^
	Control group	57	35.54±8.52	32.89±8.88^b^	30.14±7.90^bd^	24.21±7.85^bde^			
FACES-II-CV	Intervention Group	61	56.41±10.10	67.90±12.92^bc^	74.93±14.21^bcd^	82.08±17.06^bcde^	20.61^a^	319.34^a^	97.82^a^
	Control group	57	55.74±9.74	59.18±11.89^b^	61.44±13.32^bd^	63.00±14.53^bd^			

CD-RISC, Connor-Davidson Resilience Scale; HHS, Herth Hope Scale; HAMD, Hamilton Depression Rating Scale; FACES-II-CV, Family Adaptability and Cohesion Evaluation Scale, second edition; ^a^ *P*< 0.001; ^b^ *P*< 0.05, compared with Baseline; ^c^ *P*< 0.05, compared with control group at same time; ^d^ *P*< 0.05, compared with 12-week; ^e^ *P*< 0.05, compared with 24-week.

The GEE test showed that the frequency of NSSI in the control group was not significantly different from that in the intervention group (Wald = 0.55, *p* = 0.46 > 0.05, **Table 4**). In addition, the results of the repeated-measures time comparison showed that the frequency of self-injury improved in both groups after 12, 24, and 36 weeks of intervention compared to baseline (*p*< 0.05, **Table 4**).

**Table 4:** Generalized estimating equation analyses of the OSI score from baseline to post-test.

Variables	$\overset{⌢}{\beta }$	$S_{\bar{x}}$	95% Wald confidence interval		*Waldχ^2^*	*P*
			Lower	Upper		
Threshold (NSSI)=3	-0.99	0.27	-1.52	-0.45	13.13	0.000
(NSSI)=2	2.08	0.33	1.43	2.73	39.13	0.000
(NSSI)=1	4.95	0.45	4.05	5.83	118.47	0.000
Intervention group	0.27	0.37	-0.45	0.99	0.55	0.46
Control group	0^a^					
Time 1(baseline)	0^a^					
Time 2 (12-week)	1.32	0.29	0.75	1.89	20.91	0.000
Time 3 (24-week)	1.82	0.32	1.20	2.44	33.30	0.000
Time 4 (36-week)	2.19	0.35	1.51	2.86	40.08	0.000
Time 1×intervention group	0^a^					
Time 2×intervention group	2.05	0.40	1.27	2.83	26.67	0.000
Time 3×intervention group	1.87	0.39	1.11	2.62	23.43	0.000
Time 4×intervention group	2.19	0.40	1.41	2.98	26.67	0.000
Time 1×control group	0^a^					
Time 2×control group	0^a^					
Time 3×control group	0^a^					
Time 4×control group	0^a^					

a set to zero because this parameter is redundant; NSSI: Self-injurious behavior in the past month; (NSSI)=1: one act of NSSI once a month; (NSSI)=2: one act of NSSI at least once a week; (NSSI)=3: one act of NSSI once a day; time 1=baseline, time 2=12-week, time3=24-week, time 4=36-week.

## 4 Discussion

SMART is widely used in various contexts; thus far, however, it has not been used in parent–child groups for adolescents with NSSI. The training content is based on conscious, short daily practice and is applicable to daily life scenarios, emphasizing attention training and developing a core mindset to reinterpret life events and consciously reconstruct them; helping participants learn novel cognitive, emotional, and behavioral strategies; and enhancing their sense of control (24). In the current study, we compared the effect of parent–child group emotion regulation and resilience training with usual treatment in adolescents with NSSI. The results showed that CD-RISC, HHS, HAMD-24, and FACES-II-CV scores improved in the intervention group compared to the control group; this suggests that parent–child group emotional regulation and resilience training can improve depression, hope, resilience, and adaptive and cohesive family functioning in adolescents with NSSI. However, no significant difference was observed in the frequency of self-injury between the two groups. The results indicated that adherence to individual or group therapy had comparable effects to that of the intervention group on self-injurious behaviour in adolescents with NSSI.

Parent–child group interventions may be more cost-effective than family therapy and provide ample opportunities for parents and adolescents to discuss NSSI and its implications for them. Furthermore, teens and parents can benefit from the support of other teens and parents. Parents who participate tend to gain a sense of belonging to the group, which also benefits their psychological well-being. For parents who lack knowledge about NSSI, the parent–child therapy model serves as a classroom where they can gain disease-related knowledge and conflict resolution skills, which allows them to actively participate in their children’s recovery process (34). Mutual encouragement and support from parents in the group may also contribute to maintaining the intervention’s effectiveness. Furthermore, an environment with adult participation provides opportunities for social connection, socialization, and behavioral activation, which may be lacking in adolescent group therapy (35).

The psychological problem of adolescence may stem from the difficulty of the parent-child relationship or lack of security (etc.) during infancy or growth (17) so providing an environment for adolescents and parents to work together may also promote the repair of the parent-child relationship. The “gratitude and acceptance” training increases family intimacy and enables parents and children to express their love for each other, thus enhancing mutual understanding and creating a safe environment where parents and children can accept their imperfections. After excessive or unrealistic expectations are reduced, the parent–child relationship can be improved, eventually influencing NSSI behavior. This finding is consistent with a cohort study of 2,127 adolescents that explored the relationship between parent–child dynamics and NSSI, demonstrating that positive parental behaviors can reduce the incidence of NSSI (14). In addition, a harmonious family atmosphere fosters children’s resilience.

Resilience refers to an undisturbed mental health trajectory during periods of adversity or stress or successful recovery from temporary dysfunction (36). Resilience is the outcome of dynamic interactions between an individual and the environment, which can be modulated by both personal (e.g., optimism) and environmental factors (e.g., social support) and can be increased through intervention (37, 38). Although there was no statistical difference between the two groups in self-injury behavior, the reduction in HAMD score among adolescents in the intervention group may be attributed to the improvement in resilience, consistent with the findings of Seshadri et al. (37, 38). The practice of mindfulness meditation in the intervention group instructed patients and their parents to focus on the “present moment” without engaging in judgmental thinking. The cultivation of nonjudgmental thinking, focusing on the present moment and mental phenomena (e.g., physical sensations, thoughts, and emotions), along with attention training, enables patients to accept what is and helps them adapt to stress more calmly. By focusing on the present moment, one may become more aware of the positive aspects of life; therefore, mindfulness-based resilience interventions may enhance participants’ optimism or frequency of positive emotions. Conversely, learning to accept negative emotions and situations may improve participants’ cognitive flexibility (39, 40). Psychopathological theory suggests that emotional regulation dysfunction is primarily caused by mental inflexibility (41). Therefore, resilience interventions in intervention groups that adopt relaxation techniques, mindfulness concepts, and skills can improve the resilience of both adolescents and their parents, enhancing their ability to adapt to stress and fostering hope in life (42).

O’Doherty suggests that stimulating inner hope is a way to promote recovery in individuals experiencing psychological distress (43). Parent–child group therapy provides a continuous supportive environment for family members. Stressor analysis can help patients and parents become aware of the difficulties that adolescents face during this stage of life, and the parent–child interaction mode increases parents’ understanding of their children and improves their understanding of key psychosocial resources, such as inherent strengths and abilities. Alternatively, meditation also helps increase the participants’ hope levels. An intervention study with Indian college students found that meditation intervention improved students’ resilience and hope levels, and the two were positively correlated and mutually reinforcing; this is beneficial for buffering anxiety and depression and promoting positive mental health outcomes (44).

Nevertheless, no significant difference in NSSI frequency was observed between the intervention and control groups, which implies that the intervention program in this study was not more effective than the control program in reducing the occurrence of NSSI. This might be related to factors such as an insufficient sample size, short intervention duration, and lack of specificity in the intervention content of this study. However, both groups showed improved NSSI frequency after 12, 24, and 36 weeks of intervention compared to baseline. This may indicate that both regular psychological counseling and parent–child group emotion regulation and resilience training could alleviate adolescent self-injury behavior to some extent. Long-term interventions are needed to explore the long-term effects of parent–child group emotional regulation and resilience training on adolescent NSSI behavior.

Effect in intervention group persisted over time at follow-up may be due to the simultaneous involvement of parents and children, so that the family atmosphere does not change immediately with the end of treatment, as evidenced by the continuous decline in depression scores and the increase in family cohesion and hope levels. Future studies should extend follow-up to observe the long-term development and growth of adolescents and family under this intervention and supporting further clinical practice of non-pharmacological treatments for depression and NSSI in adolescents.

### 4.1 Limitations

This study had some limitations. First, we only collected data from patients and did not evaluate the parents. Future studies should analyze parental contributions to the outcomes. Second, parents who were willing to spend time with their children to complete the entire study may have been strongly motivated to find an appropriate solution, which may have caused some sample bias.

## 5 Conclusions

Parent–child group emotional regulation and resilience training is a comprehensive training model that integrates cognitive training, positive thinking, relaxation, and other psychotherapeutic techniques to reduce NSSI behaviors in adolescents by enhancing parent–child relationships and fostering resilience in both parents and children. This study applied this method to adolescents with NSSI, increasing their hope and resilience levels and improving family harmony and resilience. Although the intervention group did not differ significantly from the control group in terms of reduced NSSI frequency, both groups exhibited a significant decrease in NSSI frequency compared to baseline.

## Data availability statement

The original contributions presented in the study are included in the article/supplementary material. Further inquiries can be directed to the corresponding authors.

## Ethics statement

The studies involving humans were approved by First hospital of Shanxi Medical University. The studies were conducted in accordance with the local legislation and institutional requirements. The participants provided their written informed consent to participate in this study.

## Author contributions

JC: Conceptualization, Data curation, Formal Analysis, Methodology, Project administration, Resources, Writing – original draft, Writing – review & editing. JZ: Data curation, Formal Analysis, Writing – original draft, Writing – review & editing. BS: Conceptualization, Data curation, Resources, Writing – original draft, Writing – review & editing. HH: Data curation, Visualization, Writing – original draft, Writing – review & editing. NL: Data curation, Writing – original draft, Writing – review & editing. YC: Project administration, Visualization, Writing – original draft, Writing – review & editing. XL: Data curation, Formal Analysis, Writing – original draft, Writing – review & editing. YD: Data curation, Writing – original draft, Writing – review & editing. WB: Data curation, Formal Analysis, Writing – original draft, Writing – review & editing. ZL: Data curation, Formal Analysis, Writing – original draft, Writing – review & editing. SH: Data curation, Formal Analysis, Investigation, Project administration, Writing – original draft, Writing – review & editing.
